# Cereal products derived from wheat, sorghum, rice and oats alter the infant gut microbiota *in vitro*

**DOI:** 10.1038/s41598-017-14707-z

**Published:** 2017-10-30

**Authors:** Hasinika K. A. H. Gamage, Sasha G. Tetu, Raymond W. W. Chong, John Ashton, Nicolle H. Packer, Ian T. Paulsen

**Affiliations:** 1 Department of Molecular Sciences, Macquarie University, Sydney, NSW2113 Australia; 2 Sanitarium Health and Wellbeing, Sydney, NSW2261 Australia

## Abstract

The introduction of different nutrient and energy sources during weaning leads to significant changes in the infant gut microbiota. We used an *in vitro* infant digestive and gut microbiota model system to investigate the effect of four commercially available cereal products based on either wheat, sorghum, rice or oats, on the gut microbiota of six infants. Our results indicated cereal additions induced numerous changes in the gut microbiota composition. The relative abundance of bacterial families associated with fibre degradation, *Bacteroidaceae*, *Bifidobacteriaceae*, *Lactobacillaceae*, *Prevotellaceae*, *Ruminococcaceae* and *Veillonellaceae* increased, whilst the abundance of *Enterobacteriaceae* decreased with cereal additions. Corresponding changes in the production of SCFAs showed higher concentrations of acetate following all cereal additions, whilst, propionate and butyrate varied between specific cereal additions. These cereal-specific variations in the concentrations of SCFAs showed a moderate correlation with the relative abundance of potential SCFA-producing bacterial families. Overall, our results demonstrated clear shifts in the abundance of bacterial groups associated with weaning and an increase in the production of SCFAs following cereal additions.

## Introduction

The human gut microbiota co-develops with the host in early life^1^. Initial microbial colonisation of the gut depends on various maternal and postnatal factors such as *in utero* environment, mode of delivery (vaginal or caesarean-section), gestational age, environment, antibiotic treatments, host genetics and diet (breast milk, formula milk or solid food)^2–6^. These factors shift the composition and functions of infant gut microbiota towards an established adult-like status within the first three years of life^6^. The adult gut microbiota is relatively more stable, higher in species diversity and lower in inter-individual compositional and functional variations compared to infants^3,7^. Accumulating data suggest a link between early life gut microbial colonisation and development of diseases, such as obesity, diabetes (type 1 and 2), food allergies and inflammatory bowel disease^8–15^. Therefore, establishment of the gut microbiota during infancy and maintenance thereafter likely plays a critical role for human health^2^.

The transition to solid food contributes significantly towards the infant gut microbiota development. This introduces infants to a larger range of plant and animal polysaccharides. Due to the lack of enzymes to digest most of these complex polysaccharides, infants largely depend on the gut microbiota to digest these otherwise non-digestible carbohydrates^16^. Therefore, weaning rapidly diversifies and alters the composition of the gut microbiota towards an adult-like composition, presumably to facilitate the metabolism of changing nutrients^5^. Exposure to new nutrients also leads to altered functions in the microbiota and production of different bacterial metabolites. For instance, introduction to solid food and more xenobiotics promote the growth of bacterial species associated with carbohydrate utilisation, vitamin biosynthesis and xenobiotic degradation^3,7^. Weaning is also linked with elevated levels of bacterial metabolic end products such as short chain fatty acids (SCFAs), possibly due to the high availability of non-digested dietary fibre^7,17^.

A number of previous studies have examined changes in the infant gut microbiota in relation to transition to solid food. Weaning in general is associated with decreased proportions of *Bifidobacteria*, *Enterobacteria* and some groups of *Clostridium*, whilst it promotes the growth of *Bacteroidetes* ^3,7,18,19^. The increase in *Bacteroidetes* could be due to their ability to digest a broad range of complex polysaccharides^7^. Very few *in vivo* studies have investigated the effect of specific dietary regimens on infant gut microbiota during the weaning phase. One such study reported the effects of feeding infants with commercially available pureed meat, iron- and zinc-fortified cereals or iron-only fortified cereals on the gut microbiota^20^. Infants fed pureed meat demonstrated enriched *Clostridium* group XIVa, whilst feeding iron-only fortified cereals resulted in decreasing the abundance of *Lactobacilli* and *Bifidobacterium* and promoting the abundance of *Bacteroides* ^20^.

Utilisation of *in vitro* models of the infant gut microbiota eliminates some of the issues associated with *in vivo* studies. *In vitro* studies reduce issues with ethical restrictions and volunteer compliance, while enabling more frequent sampling and providing a simplified system to study the gut microbiota without host interference^21^. *In vitro* infant gut microbiota model systems have been employed to investigate the effect of probiotics^22^, candidate probiotics^23^, milk lipid hydrolysis products^24^, iron^25^, milk oligosaccharides^26–28^, dietary polysaccharides and prebiotics^29–31^. Addition of short-chain fructo-oligosaccharides into an *in vitro* model of infant gut microbiota resulted in an increased abundance of the genus *Lactobacillus* while reducing the proportion of coliforms^29^. Shen *et al*. observed an increase in *Bifidobacterium* and *Bacteroides* upon addition of a prebiotic mixture of fructo-oligosaccharides and galacto-oligosaccharides into an *in vitro* model of infant gut microbiota^30^.

Lack of dietary fibre in modern Western diets has been associated with changing the gut microbiota composition, functions, diversity and spatial arrangement^32–35^. Bridging this gap in dietary fibre intake is of increasing interest as a therapeutic modulation of the gut microbiota in order to improve metabolic and inflammatory health^36^. Whole grain products generally contain a high amount of dietary fibre^37^. Although, whole grain cereals are among frequently introduced first food to infants^15^, the impact of cereals on infant gut microbiota is less well studied. In adults, consumption of whole grain maize based breakfast cereal promoted the growth of *Bifidobacterium* ^38^, whilst whole grain wheat cereal increased the abundance of *Bifidobacterium* and *Lactobacillus*/*Enterococcus* groups^39^. Consumption of whole grain barley and brown rice flakes increased the microbial diversity and reduced host markers associated with inflammation and postprandial glucose levels^40^.

Given the increasing popularity of whole grain cereals as an early weaning food and the impact on gut microbiota and disease development^15^, we chose to examine the effects of whole grain-based cereal products on the gut microbiota of infants. In this work, we investigated the effect of four commercially available cereal products, Weet-Bix™, Gluten free Weet-Bix™, Bellamy’s organic baby rice cereal and Real good food-Organic baby oat cereal on infant gut microbiota and SCFAs using an *in vitro* infant gut microbiota model system.

## Results and Discussion

Samples of four commercially available cereal products (wheat, sorghum, rice and oats based) were treated using a series of pH controlled enzyme additions and a dialysis step to simulate infant digestion. Digested cereal products were introduced into an anaerobic basal medium to examine the effects of the cereal products on the infant gut microbiota. The basal growth medium without any cereal addition was run in parallel as a control, this is referred to as the no added cereal control. All cereal and control cultures were inoculated independently with fecal homogenate obtained from a healthy infant. A total of six biological samples (one each from six different infants) were analysed. Cultures were sampled at 0, 24 and 48 hours and V4 region amplicons of the 16 S rRNA gene were sequenced. A total of 21,231,850 reads were generated. After quality filtering and rarefaction 35,095 reads per each of the 270 samples were used for further analyses (270 = 6 biological samples × 3 time points × 3 technical replicates for 5 experimental groups including 4 cereal treatments and no added cereal control).

### Each biological sample had a unique initial gut microbial composition

The bacterial phyla *Firmicutes*, *Actinobacteria*, *Proteobacteria* and *Bacteroidetes* dominated the gut microbiota of all infants at 0 hours. However, the relative abundance of these phyla differed between individuals. Similar variations in the composition were observed at a family level (Supplementary Fig. S1). The relative abundance of the family *Veillonellaceae*, which is associated with milk polysaccharide digestion was significantly higher (*P* < 0.05) in breast-fed infants compared to the formula-fed (Table 1). In agreement with our observation, Fan *et al*. 2014 have found a higher abundance of *Veillonellaceae* in breast-fed infants compared to that in formula or mixed-fed infants^41^.

**Table 1:** Metadata of the six biological samples (Sample 1–6). None of the infants were given antibiotics in at least three months prior to fecal sample submission. *Egg and spinach allergies.

Biological sample	Age (months)	Frequency of breast feeding	Frequency of formula feeding	Types of solid food introduced	Medical conditions
Sample 1	5	Daily	None	Fruits, vegetables, grain, cereal, meat, eggs	None
Sample 2	5	None	Daily	Fruits, vegetables, grain, cereals	None
Sample 3	5.5	Daily	None	Fruits, vegetables	None
Sample 4	7	Daily	Daily	Fruits, vegetables, grain, cereals, meat, eggs, dairy	Food allergies*
Sample 5	9	Daily	Daily	Fruits, vegetables, grain, cereals, meat, eggs, dairy	None
Sample 6	11	None	Daily	Fruits, vegetables, grain, cereals, meat, eggs, dairy	None

The relative abundance of the family *Lachnospiraceae* was significantly higher (*P* < 0.001) in older infants (age > 6 months) compared to younger infants (age < 6 months). Samples obtained from older infants (age > 6 months) had a higher relative abundance of known plant polysaccharide digesting bacteria such as *Lachnospiraceae*, *Ruminococcaceae* and *Bacteroidaceae*. Differences in the abundance of these bacterial families in the infant gut microbiota due to age are largely in agreement with previous studies^7,16,19,42,43^.

Some bacterial families were highly variable between individuals. This is expected given that the composition of the infant gut microbiota varies depending on factors such as the mode of delivery (vaginal or caesarean section birth), usage of antibiotics, age, diet (breast milk or formula milk) and exposure to solid food^1,2,5^. The family *Coriobacteriaceae* was abundant in sample 2 (9.8%), 4 (4.1%) and 5 (14.4%) and not observed above 0.3% in other biological samples. The relative abundance of *Porphyromonadaceae* (12.2%) was high in sample 1, whilst sample 2 had a large proportion of *Enterococcaceae* (18.4%). In sample 4 *Streptococcaceae* was abundant (9%) and *Ruminococcaceae* was abundant in sample 6 (19.8%). The oldest biological sample (sample 6) showed the lowest relative abundance of *Enterobacteriaceae* (0.7%), whilst the lowest abundance of *Bacteroidaceae* (0.3%) was observed in biological sample (sample 3), obtained from an infant that had not been exposed to cereal grains.

### All cereal additions altered the gut microbial composition

**Figure 1:**
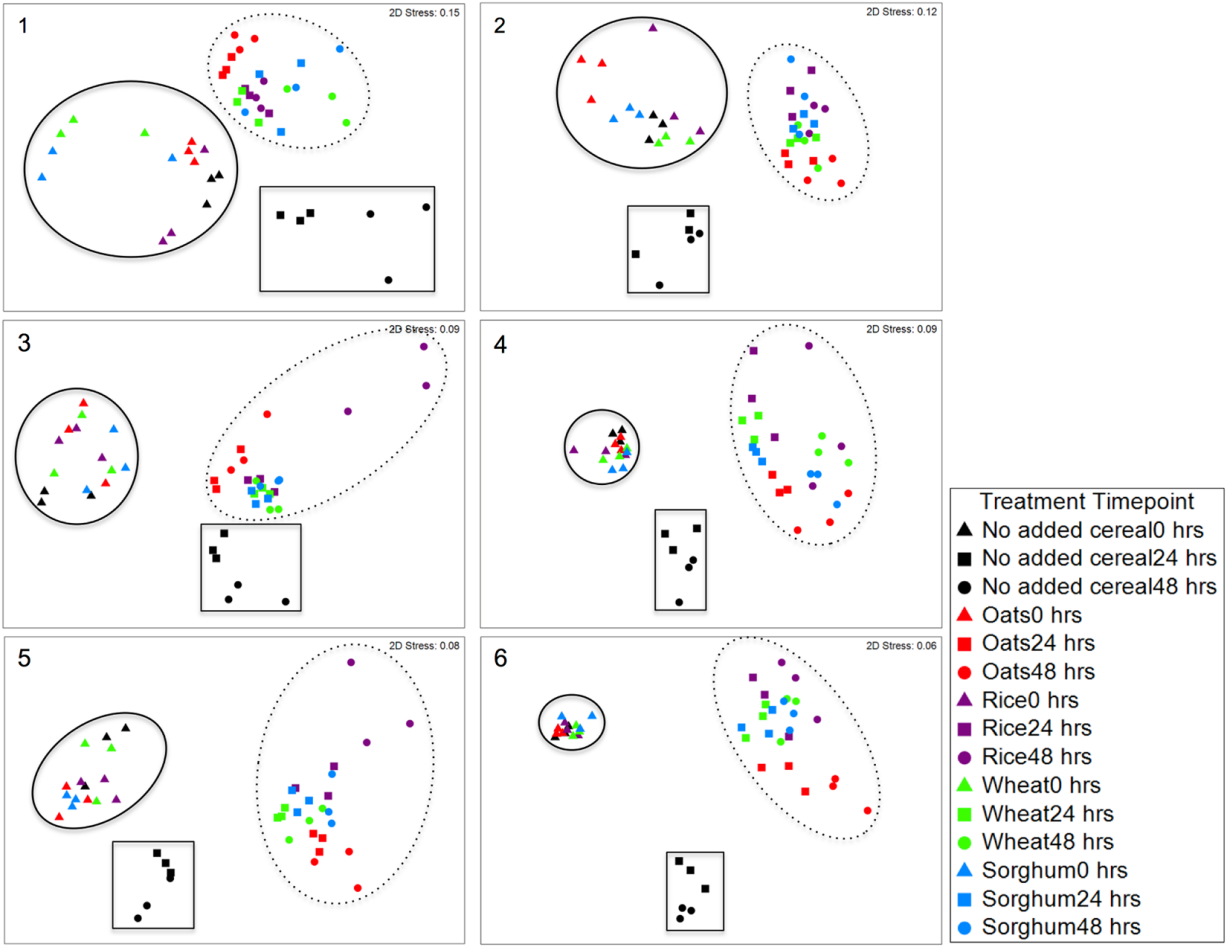
Ordination of the gut microbiota in each biological sample (1–6) at 0, 24 and 48 hours. Data is shown as Bray-Curtis similarity of Log (X + 1) transformed relative abundance based nMDS plots. Treatments and time points are colour coded as shown in the legend. All cereal additions shifted the community structure at 24 and 48 hours (dotted line circle) compared to the samples at 0 hours (solid line circle) and no added cereal control at 24 and 48 hours (solid line square).

To determine the impact of different cereal additions on the gut microbiota at 0, 24 and 48 hours, non-metric multidimensional scaling (nMDS) plots were constructed based on the relative abundances of the Operational Taxonomic Units (OTUs) (Fig. 1). Samples at 0 hours in each biological sample clustered relatively close together irrespective of the treatments. All cereal additions resulted in different microbiota community structures at 24 and 48 hours compared to the samples at 0 hours and no added cereal control at 24 hours (global analysis of similarities (ANOSIM) R > 0.7, *P* < 0.0001) and 48 hours (global ANOSIM R > 0.8, *P* < 0.0001). The microbiota community structure of the no added cereal control also changed over time, however these remained distinct from the communities after cereal addition. The cereal additions showed similar shifts to each other in the nMDS plots (Fig. 1), and consistent with this there were no statistically significant differences in the microbial community structure between the cereal products.

The bacterial diversity in each sample was determined using a Shannon diversity index. Biological sample 3 had a significantly (*P* < 0.0001) lower Shannon index (4.6 ± 0.05) at 0 hours compared to all other biological samples (Shannon diversity index of samples 1, 2, 4, 5 and 6 ranged from 4.8 ± 0.1 to 5.0 ± 0.04). The low Shannon index value in sample 3 was primarily due to the dominance of a single OTU of the common infant gut bacterium, *Veillonella dispar* (OTU 585419, relative abundance at 0 hours: 43.6% ± 7.3%). The diversity indices between the treatments were similar at 48 hours in all biological samples, except for sample 3. The diversity of this sample increased significantly at 48 hours with the addition of rice (*P* < 0.0001).

**Figure 2:**
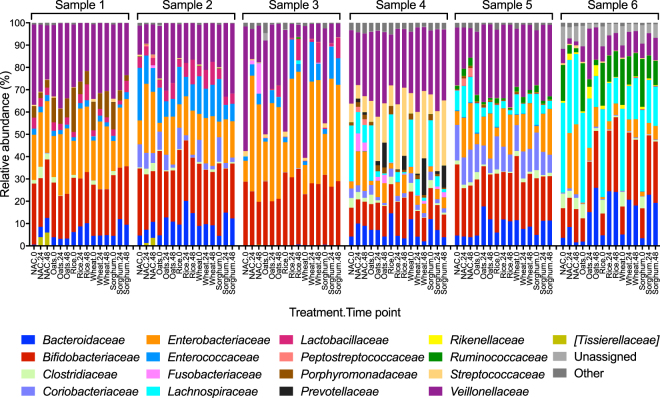
Family level taxonomic compositions of the microbial communities for each biological replicate. The relative abundances of the families were determined using QIIME and GraphPad Prism (V7). Each bar is labelled first by treatment, followed by time point (0, 24 and 48 hours). No added cereal control is abbreviated as NAC. Major bacterial families are shown in different colours as indicated in the legend. Bacterial identifications that were not assigned to a family are categorised as “Unassigned”. Bacterial families that were not significantly differentially abundant comparing the treatment regimes in any of the six biological samples are categorised as “Other”. Significance (*P* < 0.05) was determined using a Tukey’s multiple comparisons test.

The relative bacterial abundance was examined at a family level and identifications across the samples were assigned into 33 bacterial families. Statistically significant differences in family abundance across the treatments in each biological sample were investigated using a two-way analysis of variance (ANOVA) test with Tukey’s multiple comparisons test. This identified 17 families with significantly (*P* < 0.05) different abundances in at least one treatment and time point combination (Fig. 2, Supplementary Fig. S2 and Supplementary Table S2). The impact of cereal additions on the microbiota composition was highly variable between the biological samples. However, for each biological sample the relative abundance of at least one potential Carbohydrate-active enzymes (CAZymes) producing bacteria (families: *Bacteroidaceae*, *Bifidobacteriaceae*, *Lactobacillaceae*, *Prevotellaceae* and *Ruminococcaceae*) increased with addition each of the tested cereal products.

For three of the six biological samples the relative abundance of *Bacteroidaceae* increased upon addition of each of the four cereal products with the highest increase (*P* < 0.01) following addition of rice. In contrast, in biological sample 4 the relative abundance of this family significantly decreased (*P* < 0.001), while the abundance of *Prevotellaceae* significantly increased (*P* < 0.001) following all cereal additions. The families *Bacteroidaceae* and *Prevotellaceae* are members of the phylum *Bacteroidetes*, which are generally reported to degrade a wide range of dietary polysaccharides, due to their capacity to switch between energy sources depending on the availability^44–47^.

In all biological samples, the abundance of *Veillonellaceae* was significantly higher (*P* < 0.05) with addition of oats compared to all other treatments. For four out of the six samples, the abundance of this family also increased after the addition of all other cereals. Family *Veillonellaceae* is associated with utilising partial breakdown products of bacterial polysaccharide digestion and producing propionate and acetate, likely due to its limited ability to digest complex carbohydrates^18,48–51^. Previous studies have also reported an increase in the abundance of this family following *in vitro* fermentation of specific complex polysaccharides by the infant gut microbiota^27,31^.

The relative abundance of *Enterobacteriaceae* decreased following addition of each of the four cereal products, with the exception of biological sample 1, where this family significantly increased (*P* < 0.0001). According to previous studies, *Enterobacteriaceae* are usually more dominant in pre-weaned gut microbiota of younger infants and become less abundant due to weaning and age^3,19,52–54^. Therefore, the decrease in the abundance of *Enterobacteriaceae* with cereal supplementations may indicate the ability of the cereal products to aid the shift of the infant gut microbiota towards a mature status.

The relative abundance of *Bifidobacteriaceae* was significantly higher (*P* < 0.0001) with the addition of rice compared to other treatments. The family *Lactobacillaceae* was abundant in younger infants (age < 6 months) and the relative abundance significantly increased (*P* < 0.05) upon addition of rice. Previous observations of higher growth of *Bifidobacteriaceae* and *Lactobacillaceae* in the gut microbiota of adults and animal models upon addition of cereal grains^38–40^, particularly, brown rice^55–58^ are also in agreement with our results.

All four tested products have been obtained from cereal grains, which are naturally high in complex sugars such as starch, cellulose, arabinoxylans and glucofructans^59^, while oats are particularly rich in β-glucans^59^. The prevalence of *Bacteroidaceae*, *Bifidobacteriaceae*, *Lachnospiraceae* and *Lactobacillaceae* in all cereal additions is consistent with the ability of the members of these families to digest cellulose, starch and other polysaccharides^45,60^. The composition of the four tested cereal products varied in regards to dietary fibre, protein, iron, polyphenols and vitamins (Supplementary Table S1). Wheat and rice based cereal products are particularly rich in iron, which has been previously demonstrated to increase the abundance of enteropathogens in the family *Enterobactericeae* and modulate butyrate-producing bacteria^25,61,62^. While we did not observe significant changes in the abundance of the *Enterobactericeae* or butyrate-producing bacteria in samples with wheat supplementation, the abundance of potential butyrate-producing bacteria *Bacteroidaceae* and *Bifidobacteriaceae* was higher upon rice supplementation.

Biological sample 4, obtained from an infant who suffered from food allergies, showed a considerably higher initial relative abundance of *Streptococcaceae* than other biological samples. This sample displayed a notable expansion in the relative abundance of *Steptococcaceae* (*P* < 0.0001) upon all cereal additions, this family showed less than 1.0% relative abundance in other biological samples. As this is only a single individual, we cannot directly link the high abundance of the *Streptococcaceae* to the food allergies experienced by this individual. However, a high abundance of *Streptococcus* spp. in late infancy has been reported to be linked to allergic disease development^63^.

Microbial composition was also studied at OTU level and significant differences were determined using a two-way ANOVA with Tukey’s multiple comparisons test. Six OTUs that showed significantly different abundances (*P* < 0.01) between the treatments in at least three biological samples were identified (Supplementary Fig. S3 and Supplementary Table S3). All of these OTUs belonged to the families that are discussed above and showed similar trends in the relative abundances in each treatment.

### Cereal products increased SCFA production

**Figure 3:**
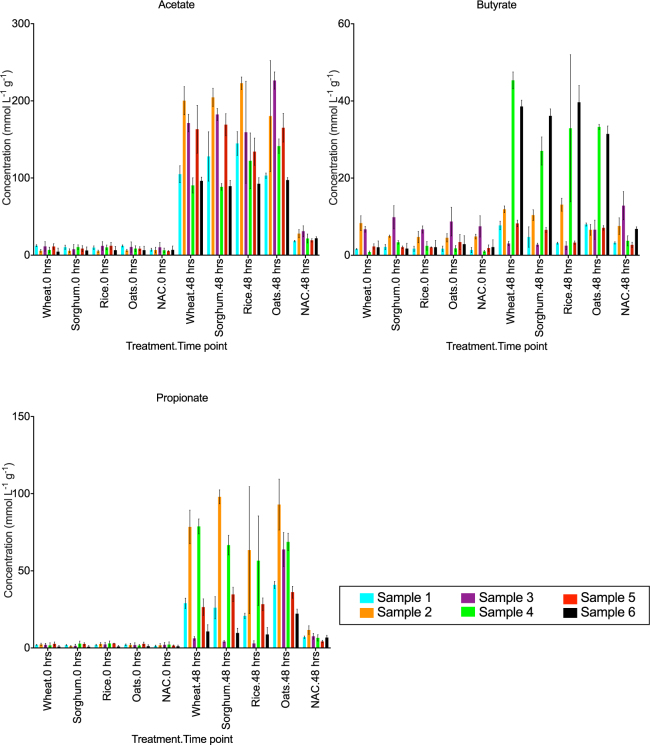
Concentration (mmolL^−1^g^−1^) of acetate, butyrate and propionate in each treatment at 0 and 48 hours. Concentration measurements at 24 and 48 hours for all three SCFAs were similar, therefore only 48 hours are shown. Mean ± SD concentration for all treatments with each biological sample (sample 1–6) denoted by colour-coded bars. No added cereal control is abbreviated as NAC. The concentrations and results of ANOVA with Tukey’s multiple comparisons test for significance are provided in Supplementary Table S4.

To investigate the effect of cereal addition on production of SCFAs, acetate, butyrate and propionate concentrations were measured from the samples collected at 0, 24 and 48 hours (Fig. 3, Supplementary Table S4). Addition of each of the four cereal products resulted in significantly higher (*P* < 0.01) concentrations of acetate across all biological samples at 24 and 48 hours compared to the no added cereal control.

Production of butyrate was higher following addition of wheat or sorghum for all biological samples except sample 3 at 48 hours. Butyrate was highly produced upon addition of rice and oats in at least four biological samples compared to the no added cereal control, however the increase in butyrate production upon supplementation with cereal products was statistically significant for only two biological replicates. Concentration of propionate was significantly higher (*P* < 0.01) in all cereal additions in biological samples 1, 2, 4 and 5 at 48 hours compared to the no added cereal control. The addition of oats significantly increased (*P* < 0.05) the concentration of propionate in biological samples 1, 3 and 6 compared to all other cereal additions.

The concentration of all three SCFAs positively correlated with the relative abundance of *Bacteroidaceae* (Spearman’s *r* = 0.21, *P* < 0.001), whilst the concentration of acetate positively correlated with *Lactobacillaceae* (Spearman’s *r* = 0.22, *P* < 0.0001) and concentration of propionate positively correlated with the relative abundance of *Veillonellaceae* (Spearman’s *r* = 0.20, *P* < 0.0001). Each of these families are known to produce SCFAs^7,27,29,31,64^. Higher production of SCFAs with cereal additions is in agreement with a number of previous studies that have also demonstrated an increase in the production of SCFAs upon gut microbial fermentation of cereal grains^65–67^. Furthermore, elevated production of SCFAs is also a characteristic weaning induced change in the infant gut microbiota during maturation to an adult-like composition^7^.

The pH of each of the culture vials with cereal additions at 48 hours showed significant reductions (*P* < 0.001) compared to the no added cereal control, which maintained the pH at the starting measurement of 7.0 ± 0.2 (Supplementary Fig. S4). Samples with rice demonstrated significantly lower (*P* < 0.001) pH levels compared to samples with oats, wheat and sorghum. pH has been previously been shown to impact gut microbiota composition, especially inhibiting the growth of pathogenic *Escherichia coli* ^68^. The metabolic activities of the major SCFA producing bacterial groups such as *Bacteroidaceae*, *Bifidobacteriaceae* and *Lactobacillaceae* have previously been reported to reduce the pH in the large intestine^69,70^. The higher abundance of at least one of these SCFA producing bacterial families and lower abundance of the family *Enterobactericeae* upon addition of all tested cereal products may be linked with the reduction in the pH.

### Predicted functional changes in response to cereal products

**Figure 4:**
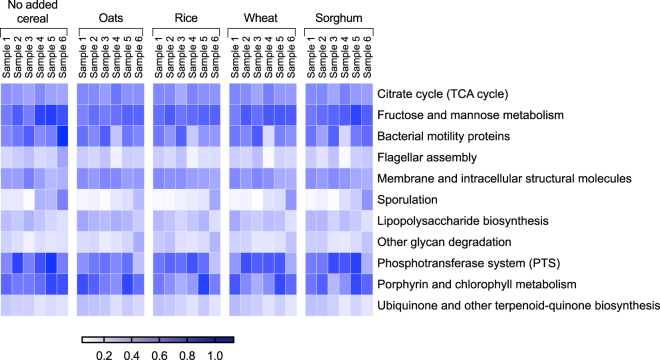
The predicted relative abundance of KEGG Orthology pathways for each sample with different cereal additions inferred using PICRUSt. The heat map shows the relative abundance of KEGG Orthology pathways (rows) with significant differences between treatments at 48 hours (columns) in at least five biological replicates. Significance was determined using an ANOVA with Tukey’s multiple comparisons test. Biological samples (Sample 1–6) were analysed independently. Blue and white represent the highest and lowest relative abundance respectively. Intensity of the colour denotes the level of the relative abundance (as shown in the legend). The inferred relative abundance of the predicted functional pathways and results of tests for significance are provided in Supplementary Table S5.

In order to investigate the effect of cereal additions on the functions of the gut microbiota, the Kyoto Encyclopedia of Genes and Genomes (KEGG) Orthology functional profiles in each treatment at 0, 24 and 48 hours were inferred from the 16 S rRNA gene abundances using phylogenetic investigation of communities by reconstruction of unobserved states (PICRUSt). This analysis predicted 12 functional pathways to be significantly differentially abundant in at least five biological samples following cereal additions (Fig. 4 and Supplementary Table S5). Based on the PICRUSt analyses, the functional category of fructose and mannose metabolism showed significantly decreased relative abundance (*P* < 0.05) in samples supplemented with oats. The Phosphotransferase system (PTS) functional category, responsible for membrane transport of simple carbohydrates, was significantly reduced (*P* < 0.01) in samples supplemented with oats, rice and wheat. There was a good positive correlation between the inferred relative abundance of the fructose and mannose metabolism functional group with the PTS functional group (Spearman’s *r* = 0.54, *P* < 0.0001), this is consistent with the primary uptake mechanism for fructose and mannose being via PTS transporters^71^. The reduction in the inferred relative abundance of these two functional pathways upon cereal addition could be linked to the addition of more complex sugars such as starch, hemicellulose, cellulose and other polysaccharides from the cereal products. Previous studies have also demonstrated a decrease in the PTS and fructose and mannose metabolism in adults and animal models upon consumption of dietary fibre^72,73^.

The inferred relative abundance of functional pathways for glycan degradation increased (*P* < 0.05) following addition of the cereal products, which could be linked to the presence of plant protein N-linked glycans, due to the availability of glycoproteins in all cereals^74^. The relative abundance of this pathway correlated with the abundance of *Bacteroidaceae* (Spearman’s *r* = 0.51, *P* < 0.0001). The correlation between glycan degradation and the family *Bacteroidaceae* is in line with the known ability of this family to digest a range of glycans^44,75^. Similar changes in these pathways have also been previously observed in animal models such as piglets, upon introduction to solid food^76^.

The inferred abundance of functional groups for lipopolysaccharide biosynthesis significantly increased (*P* < 0.05) upon addition of oats, wheat and sorghum, with the highest increase observed with the addition of oats. The inferred relative abundance of this pathway correlated with the relative abundance of the Gram-negative *Veillonellaceae* (Spearman’s *r* = 0.47, *P* < 0.0001), and negatively correlated with the Gram-positive *Lachnospiraceae* (Spearman’s *r* = −0.56, *P* < 0.0001), *Rikenellaceae* (Spearman’s *r* = −0.51, *P* < 0.0001) and *Ruminococcaceae* (Spearman’s *r* = −0.60, *P* < 0.0001). This is in agreement with the occurrence of lipopolysaccharides in Gram-negative bacterial cell wall^77,78^.

## Conclusions

We observed clear shifts in the infant gut microbiota upon addition of each of the cereal products into a large intestine simulating basal medium inoculated with a fecal sample. The relative abundance of the families *Bacteroidaceae*, *Veillonellaceae*, *Enterobacteriaceae*, *Bifidobacteriaceae*, *Lachnospiraceae* and *Lactobacillaceae* significantly changed following cereal supplementation. There were corresponding changes in the concentrations of short chain fatty acids. The concentration of acetate increased with each cereal, whilst the concentrations of butyrate and propionate significantly changed only in specific biological samples with specific cereal additions.

Supplementation with all four cereal products was observed to promote the growth of plant polysaccharide digesting bacteria, reduce the abundance of dominant families in the pre-weaned gut and increase the production of SCFAs. Therefore, these cereal products may have the potential to aid the establishment of a mature gut microbial community. Utilisation of an *in vitro* gut mimicking model system in the present study facilitated frequent sampling without host interference. However, extension of this work *in vivo* would be useful as it would eliminate the inherent limitations of batch culture gut microbiota model systems such as accumulation of bacterial metabolites due to absence of host interactions. Therefore, similar *in vivo* studies using biological samples from a narrower age range would provide further insight into the impact of cereal products on the gut microbiota and host health parameters associated with different weaning diets.

## Methods

### *In vitro* digestion of cereal products

Cereal products used in this experiment are derived from whole grain wheat (Weet-bix™), whole grain sorghum (Gluten free Weet-Bix™), organic brown and white rice (Bellamy’s organic baby rice cereal) and organic oats (Real good food-Organic baby oat cereal) (full nutritional profile and ingredient list provided in Supplementary Table S1). Weet-Bix™, Gluten free Weet-Bix™, Bellamy’s organic baby rice cereal and Real good food-Organic baby oat cereal are referred as wheat, sorghum, rice and oats cereal products, respectively, here after.

All enzymes and reagents were purchased from Sigma Aldrich, Australia, unless otherwise stated. Wheat, sorghum, rice and oat based cereal products were purchased from a local Australian supermarket.

Wheat and sorghum based cereal products were ground with a mortar and pestle under sterile conditions prior to *in vitro* digestion. Each of the four cereal products and a sterile water (MilliQ, Millipore, Australia) sample as a no added cereal control was processed through simulated oral, gastric and small intestine digestion according to published protocols^79^ with slight modifications. Lower concentrations of salivary alpha amylase (150 U/mL)^80^, gastric pepsin (3125 U/mL)^81^, small intestine bile salt (2.5 × 10^−3^ M), pancreatic trypsin (10 U/mL), chymotrypsin (2.5 U/mL), lipase (2,000 U/mL), colipase (4,000 U/mL), amylase (20 U/mL)^81,82^ and a higher level of pH (3.0) in the gastric digestion step were maintained to accommodate the differences in infant digestive system^81^. Following the digestion, cereal products were dialysed at 5 °C in a 2000 MWCO dialysis membrane (Spectra/Por 6, Spectrum Labs) against a sterile NaCl (10 mM) dialysate for 12 hours, which was followed by an additional 2 hour incubation with fresh dialysate^83^. Dialysed cereal products and the no added cereal control were frozen at −80 °C and freeze dried prior to use.

### Preparation of the basal medium

A basal medium was used which was designed to simulate large intestine conditions. The composition of the basal medium per litre was: Peptone 0.5 g, yeast extract 0.5 g, NaHCO_3_ 6 g, Hemin solution (0.05% (w/v) Hemin and 0.2% (w/v) NaOH) 1 mL, L-cysteine HCl 0.5 g, Bile salts 0.5 g, Tween 80 2 mL, Resazurin solution (0.1% (w/v)) 1 mL, Vitamin stock^84^ 1 mL, K_2_HPO_4_ 0.228 g, KH_2_PO_4_ 0.228 g, (NH_4_)_2_SO_4_ 0.228 g, NaCl 0.456 g, MgSO_4_ 0.0456 g, CaCl_2_.2H_2_O 0.0608 g and 1 mL trace mineral solution^85^ with additional NiSO_4_.6H_2_O (0.1 g/L), Na_2_SeO_4_ (0.19 g/L) and Na_2_WO_2_.2H_2_O (0.1 g/L). The pH of the medium was adjusted to 7.0 ± 0.2.

Preparation of the basal medium and subsequent culturing were performed under strict anaerobic conditions using a 25% carbon dioxide, 5% hydrogen and 70% nitrogen anaerobic chamber (Thermo Scientific model 1025 Forma). Anaerobic medium was aliquoted into airtight glass vials with rubber stoppers and aluminium lids prior to sterilisation.

### Collection and preparation of fecal inocula

All experimental procedures and protocols were reviewed and approved by Macquarie University Human Research Ethics Committee (Reference number 5201400595) and all methods were performed in accordance with the relevant guidelines and regulations. One fecal sample each was collected from six healthy infants (4 female and 2 male) aged 5–11 months. None of the infants were given antibiotics in at least three months prior to sample submission. Infants were fed breast milk (n = 2), formula milk (n = 2) or both (n = 2). All infants were exposed to solid food prior to sample collection. Four infants were introduced to a wider range of food types compared with the other two infants (Table 1).

Fresh fecal samples were collected in a sterile container and immediately placed in an anaerobic jar (Anaero jar, Oxoid Limited, UK) with an Anaerogen sachet (Oxoid) and an anaerobic indicator (Oxoid). Samples were transported anaerobically and laboratory processing was commenced in less than two hours of collection. Fecal slurries were prepared from individual samples by homogenising in anaerobic sterile basal medium and filtering through a sterile nylon mesh cloth (985 µm) prior to using as an inoculum. Fecal slurry preparation was performed under strict anaerobic conditions as used for media preparation.

### *In vitro* fermentation of the cereal products

*In vitro* digested and freeze dried samples of wheat, sorghum, rice and oats based cereals were added into separate sterile anaerobic vials with the basal medium. A control sample was run in parallel with no added cereal. The final concentration of the cereal additions was maintained at 1% (w/v). Each of these vials were then inoculated with filtered fecal homogenate to obtain a final concentration of at least 0.6% (w/v) in a final volume of 50 mL (0.3 g feces per vial). Experiments were performed in triplicate for each of the fecal samples obtained from six healthy infants. All culture vials were anaerobically incubated at 37 °C with agitation (100 rpm). Aliquots (2 mL) from these cultures were harvested at 0, 24 and 48 hours of incubation and were stored at −80 °C prior to further analyses. The pH of the cultures at 48 hours were measured using pH indicator strips universal pH 0–14 and pH 4.5–10 (Dosatest, VWR, Australia).

### Analysis of the gut microbiota

Harvested cultures were used to collect microbial cells by centrifugation at 20,238 × g for 15 minutes. Total community DNA was extracted from cell pellets using a FastDNA spin kit (MP Biomedicals) according to the manufacturer’s instructions. The lysing matrix in the kit was replaced by Lysing matrix E (MP Biomedicals)^86^. The 16 S rRNA (V4 region) gene was amplified from extracted DNA using 515 (5′-GTGCCAGCMGCCGCGGTAA-3′) forward and 806 (5′-GGACTACHVGGGTWTCTAAT-3′) reverse primers with custom barcodes^87,88^. PCR amplification, amplicon quantification, purification and sequencing using an Illumina MiSeq V4 platform (2 × 250 bp paired-end sequencing) were conducted at the Ramaciotti Centre for Genomics, Australia.

Two independent Illumina Miseq sequencing runs were performed on all samples (n = 270) as technical replicates of sequencing. Quantitative Insights Into Microbial Ecology (QIIME) software (version 1.9.1)^89^ was used to process the raw sequence data. Full length and high quality (-q 19 and with other default parameters) reads were used to determine OTUs pre-clustered at 97% similarity using an open-reference protocol against the Greengenes database (version 13_8)^90^.

After confirming the reproducibility of the two Illumina MiSeq sequencing runs, raw data for each sample were combined and reanalysed using QIIME software according to the methods described above. This resulted in a total of 21,231,850 reads (mean 78,636 ± 16,684) prior to filtering out the OTUs with less than 0.005% reads. Reads per sample were rarefied at 35,095 reads prior to statistical analyses.

### Functional prediction using PICRUSt

Functional genes in each treatment condition at 0, 24 and 48 hours were inferred from the 16 S rRNA gene sequences using PICRUSt, online galaxy version 1.1.0^91^. All *de-novo* OTUs were removed from the open-reference picked OTUs (filtered and rarefied) and those with Greengenes database (version 13_8) identifications were retained for analysis in PICRUSt. These new OTUs were normalised by the 16 S rRNA copy number and functional genes were inferred using KEGG Orthology genes^92^. The inferred KEGG Orthology genes were grouped into functional pathways at the third BRITE hierarchy level using PICRUSt. A total of 5,516,828,518 (mean 20,432,698 ± 4,553,675) KEGG Orthology genes were predicted. Each of the 270 samples was rarefied at 15,198,942 KEGG Orthology genes. Functional pathways inferred to have >10% higher/ lower relative abundance in at least one cereal addition compared to the no added cereal control were identified. Biological samples were analysed individually and the inferred functional pathways that showed >10% change in at least five biological replicates were used for further statistical analysis.

### Quantification of SCFAs

The supernatants (500 µl) of the samples collected at 0, 24 and 48 hours were spiked with an internal standard (4-methyl valeric acid). This was further diluted in a 70% (v/v) ethanol and 0.1% (v/v) trifluoroacetic acid (TFA) solution to obtain a final concentration of the internal standard in the mixture at 100 ppm. The solution was then vortexed and filtered through a 0.2 µm membrane (Millipore, Australia) prior to analysis using a gas chromatograph with a flame ionisation detector (GC-FID, Shimadzu GC-17A). Samples were separated on a 30 m × 0.25 × 0.5 µm i.d. HP-INNOWax fused silica column (Hewlett-Packard) as per the manufacturer’s instructions. GC-FID analysis for each of the 270 samples was performed with further instrument specific technical triplicates (n = 810). SCFA concentrations were normalised for the weight of the fecal inoculum in each biological sample.

### Statistical analyses

Statistical analyses of the gut microbiota sequence data were performed on filtered and rarefied OTUs using PRIMER-7 software package^93^. Non-metric multidimensional scaling (nMDS) plots were constructed based on Bray-Curtis similarity matrices of Log (x + 1) transformed abundance of the OTUs. One-way ANOSIM was performed with 9999 permutations using the Bray-Curtis similarity matrix for each biological sample. An ANOSIM R-value closer to 1 indicates a higher separation of the microbiota structure between samples, whilst R closer to 0 indicates a lower separation. The Shannon diversity index for each sample was determined based on the OTU abundance using the PRIMER-7 software package.

Bacterial families and OTUs with more than 1% relative abundance in at least three biological samples were used for further statistical analyses. Significant differences in the relative abundance of 16 S rRNA gene identifications (family and OTU level), relative abundance of inferred KEGG Orthology pathways, concentration of SCFAs, Shannon diversity indices and pH measurements between treatments were identified using GraphPad Prism (version 7) software (GraphPad Software, USA). Two-way ANOVA with Tukey’s multiple comparisons tests were employed to compare each treatment. Biological samples were analysed individually.

The correlations between the relative abundance of bacterial families, SCFA concentrations, abundance of inferred KEGG Orthology pathways and pH measurements were determined using Spearman’s correlation analyses (two-tailed test) on GraphPad Prism (version 7) software. Correlation analyses were performed between all bacterial families, SCFA concentrations, abundance of inferred KEGG Orthology pathways and pH measurements, however, results of tests where the Spearman’s correlation (*r*) was −0.2 > *r* > 0.2 are presented.

## Electronic supplementary material

Supplementary Figures

Table S1, Table S2, Table S3, Table S4, Table S5
