# Supplementary material for: Cereal products derived from wheat, sorghum, rice and oats alter the infant gut microbiota in vitro
Source: Sci Rep. 2017 Oct 30;7:14312. doi: 10.1038/s41598-017-14707-z (PMC5662621; doi:10.1038/s41598-017-14707-z)
Supplement: Supplementary file 1 — Supplementary Figures [file 41598_2017_14707_MOESM1_ESM.pdf]

Title

Cereal products derived from wheat, sorghum, rice and oats alter the infant gut microbiota *in vitro*

Authors

Hasinika K.A.H. Gamage[1], Sasha G. Tetu[1*], Raymond W.W. Chong[1], John Ashton[2], Nicolle H. Packer[1], Ian T. Paulsen[1*]

Supplementary figures and tables

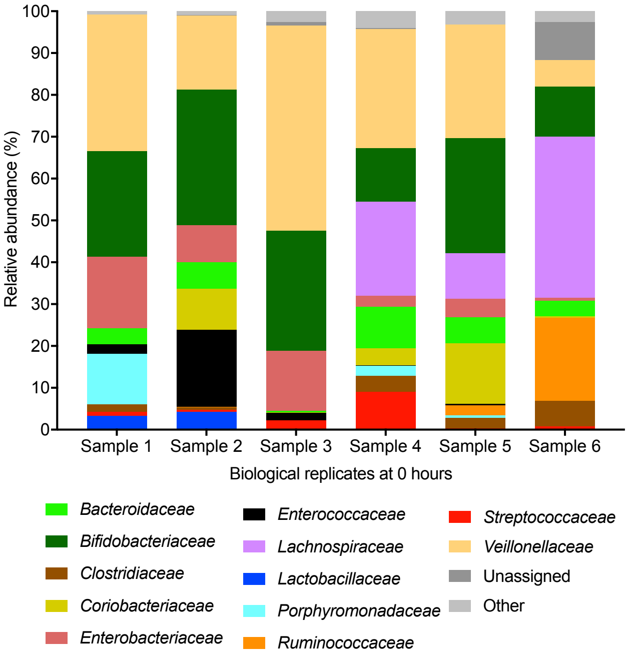

Figure S1 Family level taxonomic composition of the initial (at 0 hours) gut microbiota. The relative abundance at the family level was determined using QIIME and GraphPad Prism (V7). Bacterial identifications that were not assigned to a family are categorised as "Unassigned". Bacterial groups with less than 2% relative abundance in all biological samples (1-6) are categorised as "Other".

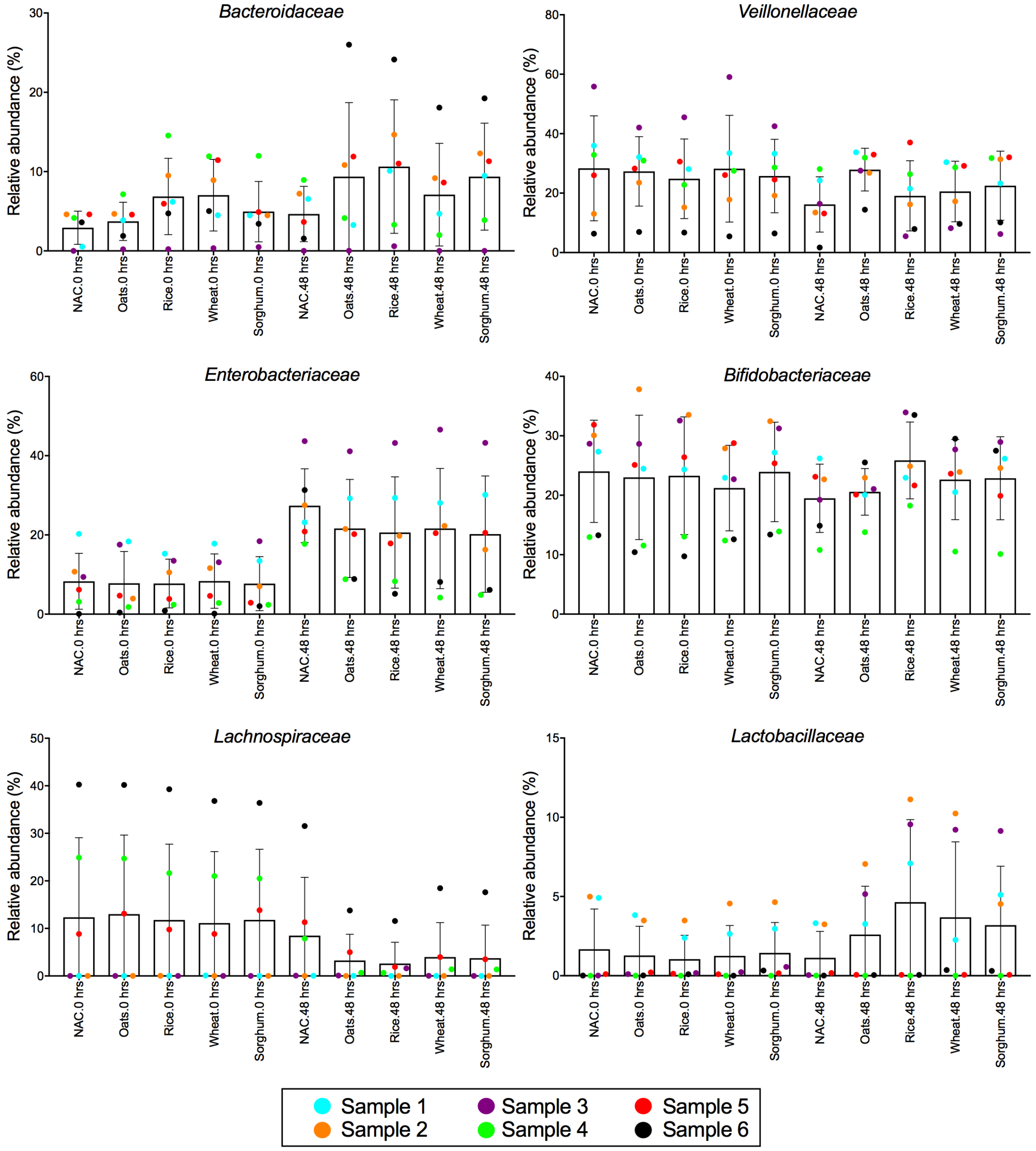

Figure S2 The relative abundance for bacterial families found to be significantly differentially abundant between treatments in at least three biological samples. Significance was determined using a Tukey's multiple comparisons test. Biological samples were analysed individually. The relative abundance of the families *Bacteroidetes*, *Veillonellaceae, Enterobacteriaceae, Bifidobactericeae, Lachnospiraceae* and *Lactobacillaceae* are shown. No added cereal control is abbreviated as NAC. In the bar graph, bars represent the mean relative abundance of all biological samples for each treatment and time point with ± SD. Mean relative abundance for each biological sample (sample 1-6) is denoted by colour-coded dots as shown in the legend. The relative abundance of these families and results of tests for significance are provided in Supplementary Table S2.

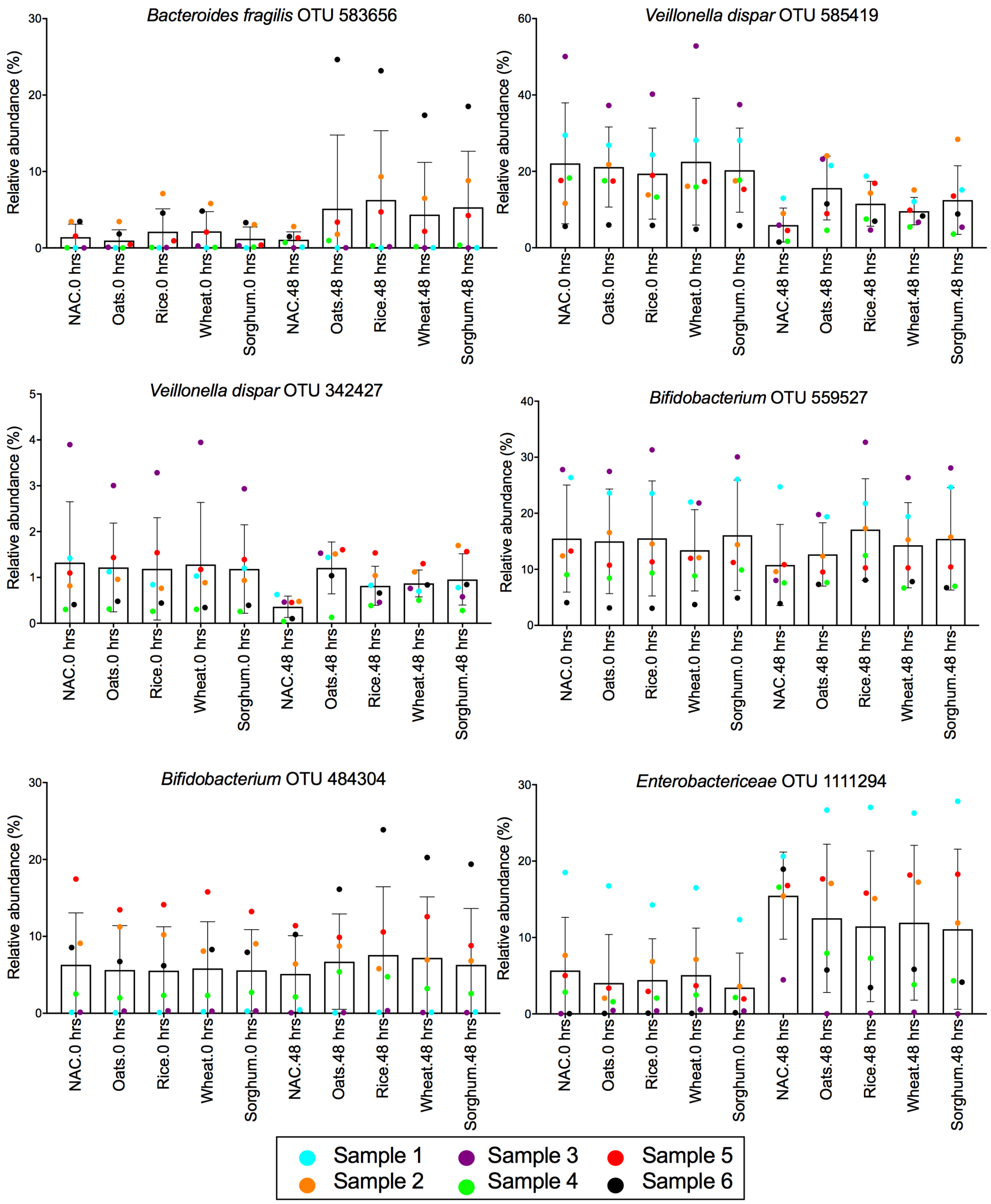

Figure S3 The relative abundance of OTUs found to be significantly differentially abundant between treatments in at least three biological samples. No added cereal control is abbreviated as NAC. Significance was determined using an ANOVA with Tukey's multiple comparisons test. Biological samples (sample 1-6) were analysed independently. Mean values with ± SD are mentioned. The relative abundance of the OTUs and results of tests for significance are provided in Supplementary Table S3.

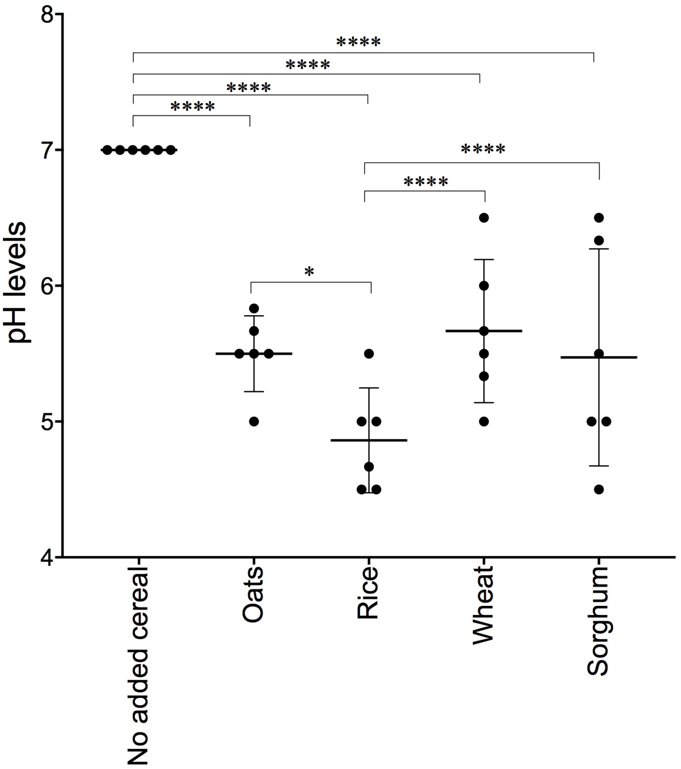

Figure S4 Measurements of pH for all cultures at 48 hours. Mean pH values for each of the three technical replicates in each of the six biological samples are indicated (dots). Bars represent the mean pH levels with ± SD for each treatment. Significance was determined using ANOVA with Tukey's multiple comparisons test (**** $P < 0.0001$).

Supplementary tables

Table S1 Nutritional information and ingredients of Weet-Bix, Gluten free Weet-Bix, Bellamy's organic baby rice cereal and Real good food-Organic baby oat cereal. NP-not provided.

Table S2 The relative abundance of bacterial families that were found to be significantly differentially abundant in at least three biological samples. Mean ± SD for each treatment and time point (0 and 48 hours) for biological samples (sample 1-6) are provided. Significance was determined using ANOVA with Tukey's multiple comparisons tests. * $P < 0.05$, ** $P < 0.01$, *** $P < 0.001$, **** $P < 0.0001$ and ns- not significant for each cereal addition compared to the no added cereal control at 48 hours.

Table S3 The relative abundance of bacterial OTUs found to be significantly differentially abundant and with more than 1% relative abundance in at least five biological samples. Mean ± SD for each treatment and time point (0 and 48 hours) for biological samples (sample 1-6) are provided. Significance was determined using ANOVA with Tukey's multiple comparisons tests. * $P < 0.05$, ** $P < 0.01$, *** $P < 0.001$, **** $P < 0.0001$ and ns- not significant for each cereal addition compared to the no added cereal control at 48 hours.

Table S4 Concentration of acetate, butyrate and propionate in each treatment at 0, 24 and 48 hours. Mean concentration per treatment for each biological sample (Sample 1-6) with ± SD is provided. Significance was determined using ANOVA with Tukey's multiple comparisons

tests. * $P < 0.05$, ** $P < 0.01$, *** $P < 0.001$, **** $P < 0.0001$ and ns- not significant for each cereal addition compared to the no added cereal control at 48 hours.

Table S5 The predicted relative abundance of KEGG Orthology pathways inferred using PICRUSt for each biological sample (sample 1-6) with different cereal additions. Mean ± SD for each treatment and time point (0 and 48 hours) are provided. Significance was determined using ANOVA with Tukey's multiple comparisons tests. * $P < 0.05$, ** $P < 0.01$, *** $P < 0.001$, **** $P < 0.0001$ and ns- not significant for each cereal addition compared to the no added cereal control at 48 hours.
